# Correlation of Clinical Outcomes with the Prominent Indication of Transcatheter Paravalvular Leak Closure: A Multicenter Experience

**DOI:** 10.3390/jcm12124047

**Published:** 2023-06-14

**Authors:** Thekla Lytra, Konstantinos Kalogeras, Theodoros Pesiridis, Carmen Moldovan, Michael Andrew Vavuranakis, Georgia Vogiatzi, Evaggelos Oikonomou, Petros Dardas, Michail Chrissoheris, Vlasis Ninios, Manolis Vavuranakis

**Affiliations:** 1 3rd Department of Cardiology, University of Athens, Sotiria Hospital, 11527 Athens, Greece; kalogerask@yahoo.gr (K.K.); carmen75mol@yahoo.com (C.M.); mikevavuranakis@gmail.com (M.A.V.); gvogiatz@yahoo.gr (G.V.); boikono@gmail.com (E.O.); vavouran@otenet.gr (M.V.); 2 1st Department of Cardiology, University of Athens, Hippokration Hospital, 11527 Athens, Greece; 3 Cardiology Department, Aghios Loukas Hospital, 55236 Thessaloniki, Greece; pdardas@otenet.gr; 4 Cardiology Department, Hgeia Hospital, 15123 Athens, Greece; mchrissoheris@hotmail.com; 5 Cardiology Department, Interbalkan Medical Center, 55535 Thessaloniki, Greece; vninios@gmail.com

**Keywords:** paravalvular leak, transcatheter closure, heart failure, hemolysis, aortic valve, mitral valve

## Abstract

Background: A paravalvular leak (PVL) is a complication following valve replacement, which may lead to heart failure and hemolysis. The aim of this study is to investigate whether the clinical outcome after transcatheter PVL closure differs according to the prominent indication of the procedure (symptoms of heart failure or hemolysis). Methods: The data of consecutive patients who had transcatheter treatment for PVL between July 2011 and September 2022 in five Greek centers were analyzed. The primary endpoint was the technical, and clinical success rates with regards to the prominent indication of paravalvular leak closure. The secondary endpoints included the evaluation and comparison of the clinical and technical success in relation to the type of valve that was treated (aortic or mitral) as well as the survival analysis in relation to the closure indication and type of valve that was treated. Results: In total, 60 patients were retrospectively studied (39% men, mean age 69.5 ± 11 years). Regarding the primary outcomes, the technical success in patients mainly suffering from hemolysis was 86.1%, while in those presenting heart failure it was 95.8%, *p* = 0.387. Furthermore, the clinical success was 72.2% and 87.5% among hemolysis and heart failure patients, respectively, *p* = 0.210. During the follow-up period, the two-year survival rates were significantly better for patients treated for the aortic valve (78.94%) compared to those in the mitral position (48.78%), *p* = 0.014. In total, 25 patients died (41.7%) during 24 months of follow-up. Conclusions: Transcatheter paravalvular leak closure can be performed with high technical and clinical success rates without any difference according to the prominent indication of closure.

## 1. Introduction

A paravalvular leak (PVL) is a major complication after surgical or transcatheter valve replacement. PVLs are the result of an incomplete seal between the sewing ring of the prosthetic valve and native annulus. They occur in 7–17% of mitral valve replacements, 5–10% of aortic valve replacements [1,2], and up to 25% of transcatheter-implanted aortic valves [2]. PVL risk factors for surgically treated patients include mechanical valve implantation, annular calcification, infectious endocarditis, and previous valve surgery [2]. Regarding post-TAVI patients, the main predisposing factors are annular calcification, a low implantation depth, and incorrect valve sizing before the procedure [3].

Heart failure and/or hemolysis are the main clinical symptoms/signs found among patients with PVL (1–3%), although a lot of patients may remain asymptomatic for a long time. When these clinical findings exist, and the patient is a high surgical risk, there is an indication for an interventional approach [4]. According to ACC/AHA and ESC guidelines, transcatheter PVL closure arose as an effective alternative therapy for patients with prosthetic valves and symptomatic HF (New York Heart Association functional class III–IV) and/or persistent hemolytic anemia, who have anatomic features that are suitable for percutaneous treatment in centers of expertise [5,6].

In this study, we retrospectively and prospectively gathered data of patients who had undergone a transcatheter PVL closure in order to determine if the clinical outcomes differ according to the main intervention indication (symptoms of heart failure or hemolysis).

## 2. Materials and Methods

### 2.1. Study Population

The present study included data from consecutive patients who had transcatheter treatment for PVL in five Greek centers (Sotiria Hospital (Athens), Hippokration Hospital (Athens), Hygeia Hospital (Athens), Aghios Loukas Hospital (Thessaloniki), and Interbalkan Medical Center (Thessaloniki)) from July 2011 to September 2022. No specific exclusion criteria were applied.

All data were collected by local investigators, anonymized, and entered into a dedicated combined PVL database, including variables of baseline clinical, imaging (echocardiographic, multi-slice, and computed tomography (CT)), and procedural characteristics as well as short (in hospital) and midterm outcomes. The local heart team of each hospital (comprising interventional cardiologists, cardiac surgeons, and imaging cardiology consultants) decided on the transcatheter treatment as well as the technical details and access route for each procedure.

### 2.2. Endpoints and Definitions

The primary endpoint of this study was defined as the technical and clinical success rates of the applied treatment with regards to the primary indication of PVL closure (symptoms of heart failure or hemolysis). The secondary endpoints were defined as the evaluation and comparison of clinical and technical success in relation to the type of valve that was treated (aortic or mitral) as well as the survival analysis in relation to the closure indication and the type of valve that was treated.

Technical success was defined as successful deployment and implantation of closure device without or with a trace of residual paravalvular leak. Clinical success was defined as an improvement of New York Heart Association (NYHA) class by one or more of the functional classes and/or an improvement of hemolysis that allowed the patient to become transfusion free for at least 6 months. Hemolytic anemia was defined by clinical and laboratory evidence of anemia (hemoglobin <15 mg/dL in men and <13 mg/dL in women, lactate dehydrogenase >500 U/L, indirect bilirubin >1.2 mg/dL) while other probable causes were excluded (e.g., hemorrhage). Heart failure symptoms were assessed according to the NYHA functional class. The clinical follow-up was evaluated either by telephone or physical visit.

### 2.3. Transcatheter PVL Closure Techniques

The percutaneous technique utilized for PVL treatment varied according to the type of valve that was treated. For mitral valve, both anterograde transeptal and retrograde transapical access were used. When the transeptal approach was not achievable, transapical access was used for crossing the mitral valve defect and implanting closure device. Transesophageal echocardiography was used to guide and confirm device implantation and adequate reduction in perivalvular regurgitation as well as to confirm normal prosthetic leaflet motion before final closure device release [7]. For aortic valve, a retrograde femoral artery approach was most commonly used. Transthoracic echocardiography may be adequate to image the leak; however, for posterior leaks, transesophageal echocardiography or intracardiac echocardiography may be needed [7,8]. On rare occasions, when more stable rail is needed, an arterio-apical loop through simultaneous apical and femoral access could be used.

As first-line periprocedural imaging, transthoracic echocardiography (TTE) was used for the assessment of severity and the anatomic location of PVL. A 3-class grading (mild, moderate, and severe) was used for both aortic and mitral valves based on criteria including LV size, regurgitant jet features, vena contracta width, circumferential extent, and diastolic pressure half-time. Clinically significant PVL was defined as at least moderate (moderate–severe) with symptoms of heart failure and/or hemolysis. Transesophageal echocardiography (TOE) was utilized for a thorough periprocedural PVL assessment and procedural guidance. Cardiac CT was used as an additional imaging modality for mapping the paravalvular defect (size, shape, and exact location) and its correlation with surrounding structures [3,4,9].

### 2.4. Device Selection

The type and number of closure devices were chosen based on the specific shape and size of the defect, aiming for its adequate and effective sealing without compromising the function of the prosthetic valve leaflets. The Amplatzer family of devices represent the main devices that were used, including the Amplatzer vascular plug (AVP) family (AVP II, AVP III, and AVP IV), the Amplatzer duct occluder (ADO I and ADO II), the Amplatzer atrial septal occluder (ASO), and the Amplatzer muscular ventricular septal defect occluder (AMVSDO). The AVP III is the only device accepted for PVL closure and holds a European conformity (CE) mark. The Occlutech Paravalvular leak device (PLD) (Occlutech, GmbH, Jena, Germany) is only available in Europe and is the first to be specifically designed for PVL closure [10,11,12,13].

### 2.5. Statistical Analysis

All continuous variables were tested for normality using the Kolmogorov–Smirnov and Shapiro–Wilk tests. Continuous variables are presented as means (± SD) and categorical variables as frequencies and percentages. In the case of skewed distribution, variables are shown as medians (interquartile range). Student’s *t*-test and Wilcoxon rank sum test were used for parametric and nonparametric continuous variables, respectively. Chi-square analysis or Fisher’s exact test was used to compare categorical variables. *p*-values ≤ 0.05 were considered statistically significant; a 95% confidence interval was used. Survival was assessed using Kaplan–Meier curves. All statistical analyses were performed with SPSS software, Version 20 (IBM Corp., Armonk, NY, USA).

## 3. Results

### 3.1. Patient and Procedural Characteristics

The data of 60 patients (mean age 69.5 ± 11 years old, 39 men) from July 2011 to September 2022 were included in the analysis. The mean duration of the follow-up time was 24 months. The prominent indication of PVL closure was hemolysis in 36 patients (60%) and heart failure symptoms in 24 patients (40%). The baseline demographic and clinical characteristics of the total population and these two subgroups are shown in Table 1. The major comorbidities of the total population included chronic kidney disease (35%), coronary artery disease (23.3%), previous CABG (20%), pulmonary hypertension (48.3%), diabetes mellitus (18.3%), and previous endocarditis (16.7%).

For the majority of the variables, the baseline demographics were similar between the two groups (hemolysis and heart failure), with the exceptions of the type of valve that was treated and a history of chronic kidney disease. Thus, hemolysis was more often the primary indication for transcatheter treatment among those treated in the mitral valve position (73.2%, *p* = 0.034), while heart failure symptoms were the main indication for those treated in the aortic position (68.4%, *p* = 0.030) (Table 2). The hemolysis group more frequently had a history of chronic kidney disease than the heart failure group (44.4% vs. 20.8%, respectively, *p* = 0.011).

**Figure 1:**
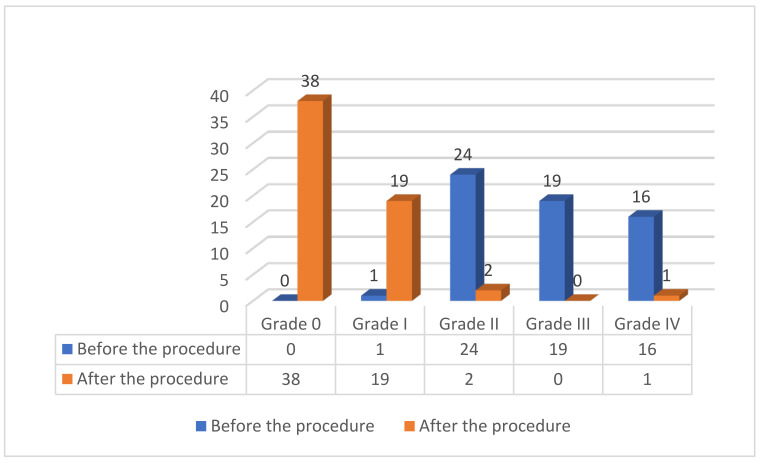
Grade of paravalvular leak before and after the procedure.

**Figure 2:**
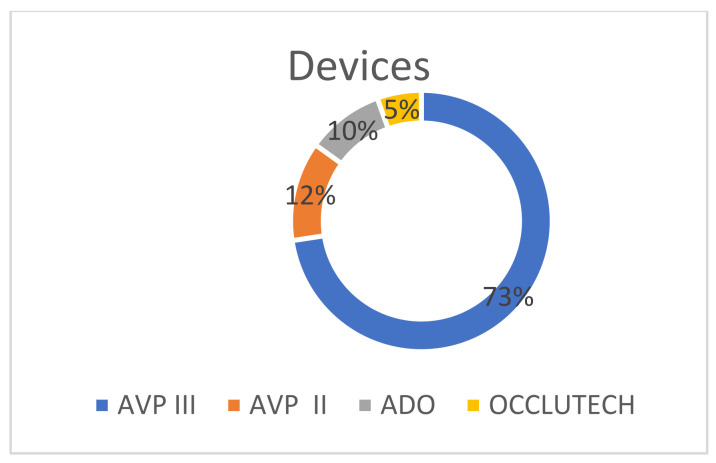
Types of used devices.

**Figure 3:**
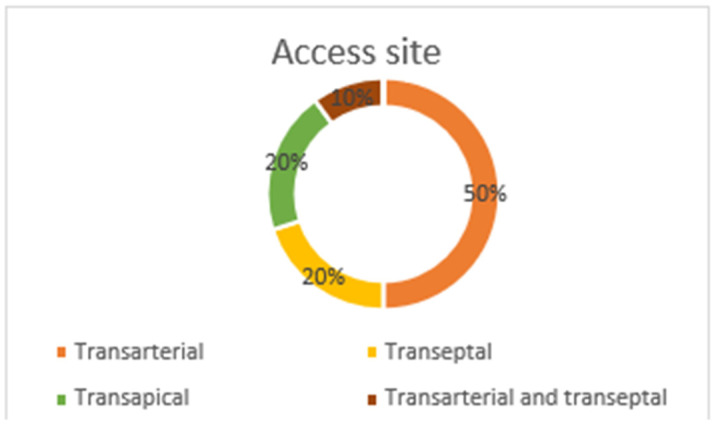
Access site.

PVL closure was performed in the position of the aortic valve in 19 patients (31.7%) and in the mitral valve in 41 patients (68.3%). In detail, for the aortic valve, most of the leaks were located in the non-coronary cusp (73.7%) and in the right coronary cusp (26.3%). For the mitral valve, the leaks were mainly lateral (58.5%), medial (34.1%), posterior (4.9%), and anterior (2.4%). The grade of PVL severity as assessed by TOE for the study cohort was grade 0 (0 patients), grade I (1 patient, 1.7%), grade II (24 patients, 40%), grade III (19 patients, 31.7%), and grade IV (16 patients, 26.7%) before the procedure and was grade 0 (38 patients, 63.3%), grade I (19 patients, 31.7%), grade II (2 patients, 3.3%), grade III (0 patients), and grade IV (1 patient, 1.7%) after the procedure (Figure 1. All patients included in the analysis were surgically treated, and none of them received a transcatheter aortic implanted valve (TAVI). In total, 73 closure devices were implanted, including 53 Amplatzer Vascular Plug IIIs, 9 Amplatzer Vascular Plug IIs, 7 ADOs, and 4 Occlutech PLDs (Table 3, Figure 2). In four patients, the device implantation was not feasible due to anatomical characteristics. Regarding the access site, transfemoral arterial access was used in 30 patients, anterograde transeptal access was used in 12 patients, and retrograde transapical access was used in 12 patients, while both transfemoral arterial and transeptal access were used in 6 patients (Table 3 and Figure 3).

### 3.2. Outcome

After the procedure, the rates of at least a moderate residual PVL (89.1% at baseline vs. 5% at discharge) and the functional status (mean NYHA class 2.88 ± 0.77 on admission and 1.57 ± 0.77 at follow-up) were statistically significantly improved, *p* < 0.001. In addition, in the whole group of patients the indexes were significantly improved 1 month after the procedure (hemoglobin 10.38 ± 1.9 vs. 11.23 ± 1.81 g/dL, *p* = 0.01; lactate dehydrogenase 1107 ± 593 vs. 611 ± 340 IU/L, *p* = 0.003; indirect bilirubin 2.34 ± 2.35 vs. 1.63 ± 1.94, *p* = 0.006).

For the total study population, the rates of technical and clinical success were 90% and 78.3%, respectively. Regarding the primary outcome, the technical success rate for patients mainly suffering from hemolysis was 86.1%, while the technical success rate for those mainly suffering from heart failure symptoms was 95.8%, indicating no statistically significant difference (*p* = 0.387). Similarly, the clinical success rates were 72.2% and 87.5% for hemolysis and heart failure patients, respectively (*p* = 0.210).

Further analysis showed that regarding the hemolysis group, for those treated in the mitral position (30 patients), the location of the leak was statistically significant correlated with the technical success rates (100% success for the anterior, lateral, medial, and posterolateral positions vs. 88.9% success for the posterior position, 50% for the anterolateral position, and 0% for the posteromedial position, *p* = 0.034). For the same group, clinical success was 100% for the posterior, lateral, posterolateral, and posteromedial positions and 50% for the anterior, medial, and anterolateral positions (*p* = 0.107). Regarding the aortic valve, no strong correlation was found between the location of the leak and the success rate. Similarly, for patients treated because of heart failure symptoms, both the clinical and technical success rates were independent of the location of the PVL for both the aortic and mitral valves.

As concerned, the primary endpoint, the clinical and technical success rates did not statistically differ among hemolysis group patients according to the grade of the PVL. In detail, the clinical success rates were 100% for grade I, 50% for grade II, 75% for grade III, and 100% for grade IV, *p* = 0.219, while the technical success rates were 100%, 71%, 58%, and 88%, respectively, *p* = 0.386. Similarly, no difference was detected among heart failure patients for clinical success (100% for grade I, 90% for grade II, 67% for grade III, and 100% for grade IV, *p* = 0.310) or technical success (100% for grade I, 100% for grade II, 83% for grade III, and 100% for grade IV, *p* = 0.372)

Regarding the secondary outcome, the technical success rates were shown to be non-significantly different for the aortic and mitral valves (89.5% vs. 90.2%, respectively, *p* = 0.926). Similarly, regarding the clinical success, the corresponding rates were 78.9% and 78% for the aortic and mitral valves, respectively (*p* = 0.937).

As regards, the secondary endpoint, the clinical and technical success in either the aortic or mitral valve did not differ according to the PVL grade. Particularly, for the aortic valve, the procedure success rate for grade I was 100%, for grade II 100%, for grade III 67%, and for grade IV 100% (*p* = 0.212), while the clinical success rates were 100%, 89%, 50%, and 100%, respectively (*p* = 0.243). Accordingly, for the mitral valve, the procedure success rates were 100% for grade I, 100% for grade II, 62% for grade III, and 89% for grade IV (*p* = 0.331). Similarly, the clinical success rates for the same valve position were 67%, 100%, 87%, and 100%, respectively (*p* = 0.341).

### 3.3. Survival

**Figure 4:**
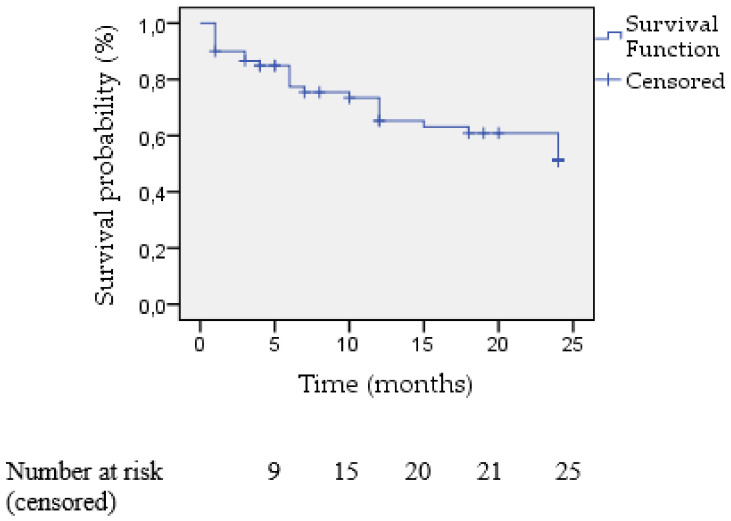
Kaplan-Meier survival in total patients in mean follow up of 24 months.

**Figure 5:**
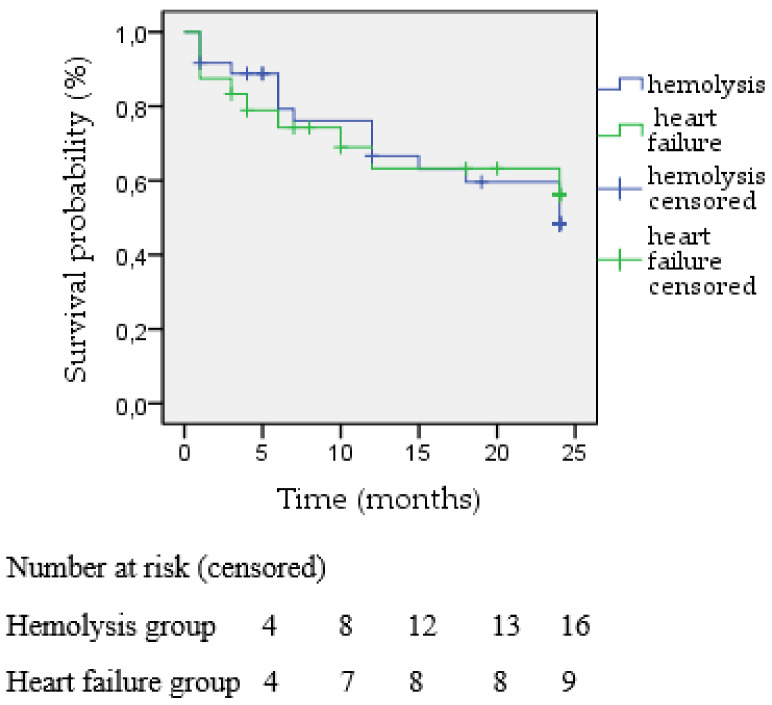
Kaplan-Meier survival stratified by the main indication of PVL closure in mean follow up of 24 months.

**Figure 6:**
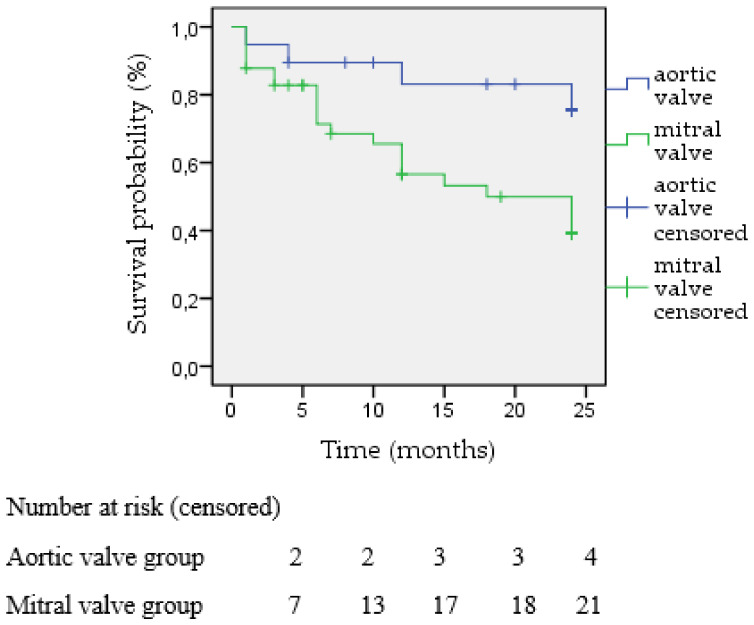
Kaplan-Meier survival stratified by the type of valve treated in mean follow up of 24 months.

During the 24-month follow-up period, 25 patients died (41.7%). The total population survival rate at 6 months was 78.3% (47 patients), at 12 months 68.3% (41 patients), and at 24 months 58,3% (35 patients) (Figure 4). The two-year Kaplan–Meier estimated survival was similar between the hemolysis and heart failure groups (55.6% vs. 62.5%, respectively, *p* = 0.906) (Figure 5). However, the two-year survival rates were statistically significantly better when treating the aortic valve (78.94%) in comparison to the mitral position (48.78%), *p* = 0.014 (Figure 6).

The main causes of death during the first 6 months of follow-up were not directly attributed to the underlying PVL and included myelodysplastic syndrome (three patients), septic shock-infection (two patients), end stage kidney disease (three patients), complications of hip fracture surgery (two patients), suicide (one patient), and jaundice due to liver dysfunction (one patient).

## 4. Discussion

This study investigates any correlation between the indication of PVL closure (heart failure symptoms or hemolysis) and clinical outcomes, including the prespecified outcomes of technical and clinical success [14]. In a total cohort of 60 patients, the rates of technical and clinical success were 90% and 78.3%, respectively.

When analyzing further in regards to the primary indication of the transcatheter closure, it was shown that no statistically significant difference exists either for technical or clinical success. This was similarly shown when investigating the same outcomes for the two subgroups of patients treated in the aortic or mitral valve position. However, the two-year survival Kaplan–Meier curves were found to demonstrate better survival rates for patients treated in the aortic valve compared to patients treated in the mitral valve.

In 1992, Hourihan et al. published the first clinical study with a catheter-based PVL closure [15]. Since then, percutaneous PVL closure techniques have been increasingly refined over the years, with two purpose-specific devices approved for transcatheter closure (AVP III and Occlutech PLD). However, there are limited studies addressing the selection of a percutaneous approach to PVL [16,17,18]. Nonrandomized trials comparing surgical with percutaneous treatment were conducted and showed similar outcomes [18]. In the HOLE registry, the technical success rate was 87% (defined as the successful delivery of a PVL closure device without interference with the valve prosthesis), and the procedural success rate was 73% (defined as the technical success of a PVL closure device and one or more grades of regurgitation reduction) [19]. In a registry from the United Kingdom and Ireland, a statistically significant improvement of the NYHA class from 2.7 ± 0.8 before the procedure to 1.6 ± 0.8 over a median follow-up of 110 days was shown, while death was reported in 16% of patients during the follow-up [20]. The aforementioned published data are in line with the results of our cohort study regarding the very promising technical and clinical success rates. The current guidelines of the American College of Cardiology (ACC), American Heart Association (AHA), and European Society of Cardiology (ESC) suggest that the percutaneous repair of paravalvular regurgitation is reasonable in patients with prosthetic heart valves and intractable hemolysis or NYHA class III/IV HF who are at a high risk for surgery and have anatomic features suitable for catheter-based therapy, when performed in centers with expertise in the procedure [5,6].

Similar to previously published studies, a variety of often off-label closure devices were also used in our cohort. Among them, both AVP III and Occlutech PLD were CE-marked for dedicated PVL closure. There is a wide range of PVLs with asymmetrical geometry, so their closure is a major challenge. Therefore, the operator should choose the appropriate type, number, and size of closure devices based on the anatomical characteristics of the defect, aiming for its adequate and effective sealing without compromising the function of the prosthetic valve leaflets. Generally, the Amplatzer family of devices represent the main apparatus used in the majority of cases in our study. Specifically, the AVP III was the most used device, mainly because it has an asymmetrical oblong shape, is available in different sizes, and encompasses a flexible nitinol waist that fits in a wide range of non-regular PVLs. Similarly, in a recent prospective registry by Hascoet et. al, AVP III was the device mostly used [21]. However, a study investigating midterm procedural and clinical outcomes when using the Occlutech device showed associations with significant clinical improvement and relatively low rates of serious complications, despite the required larger diameter sheaths for PLD devices [12].

In our study, transcatheter PVL closure was performed with high technical and clinical success rates in the total population, without any statistically significant difference between the hemolysis and heart failure groups. Indeed, when investigating the technical success rates, this finding could be reasonable, as the proper implantation of the closure device without any valve interference can be achieved irrespective of the underlying clinical condition of the patient (heart failure or hemolysis). The technical success rates can be attributed to the operators’ experience (including interventional and imaging cardiologist cooperation) and the demanding learning curve required for these procedures. However, for the clinical success rates, it seems, according to our study findings, that the successful and effective sealing of the defects leads to both hemolysis and functional status improvement. This could be due to the rapid elimination of the prosthetic valve regurgitation, leading to the gradual improvement of heart failure symptoms and immediate improvement of hemolysis due to the sealing of the defect. Interestingly, Hascoet et al. showed in a prospective registry that hemolytic anemia was associated with the absence of clinical success [21]. Indeed, when treating patients with hemolysis and PVL, the final sealing of the defect should be effective enough, as even a smaller residual leak during the initial sealing can lead to the worst hemolysis.

During the two-year follow-up period, the total survival rate was recorded as marginally above 50%. This finding is not far away from the findings of similar studies; however, it is quite indicative of the critical clinical condition of these patients along with the multiple comorbidities that they have. This high-risk population mainly suffers from major comorbidities such as chronic kidney disease, coronary artery disease, previous CABG, pulmonary hypertension, diabetes mellitus, and previous endocarditis. The main causes of death during the first 6 months of follow-up were not directly attributed to the underlying PVL and included causes related to the medical histories of the patients and other extra cardiac factors.

Our survival analysis demonstrated no difference between those suffering from hemolysis or heart failure, while the survival curve was significantly better for patients treated in the aortic position compared to those in the mitral position. This could be possibly attributed to the higher logistic Euroscore depicted in the mitral valve group (Table 2) as well as the additional comorbidities identified among these patients, including chronic kidney disease, pulmonary hypertension, and previous coronary artery bypass rates. Furthermore, mitral valve PVL treatment is more frequently completed through transapical access, which is a more invasive and complex procedure that inevitably could be the main factor contributing to the lower survival rates. In addition, as shown, most of the aortic valve patients had heart failure symptoms as the prominent indication for paravalvular leak closure, feasibly contributing to the worst survival rates. However, this finding is subject to the limited overall number of subjects included in this study, so safe conclusions cannot be reached.

Undoubtedly, transcatheter PVL closure is a field of structural heart disease treatment that is going to see major growth in the coming years. The development of new devices, the gradual improvement of operators’ skills, and the enhancement of imaging techniques are factors that will optimize the results of the technique. Inevitably, any future research efforts should be in the direction of optimizing closure devices and techniques and finding PVL patients with either hemolysis or heart failure symptoms who are going to benefit the most from a possible PVL closure. The present study, despite its limited population number, might have an application in daily clinical practice by further supporting the transcatheter treatment of PVL patients based on the high clinical and technical success rates achieved in this cohort. In addition, it seems that all PVL patients, irrespective of the initial clinical closure indication (hemolysis or heart failure symptoms), demonstrate equally high procedural success rates and similar midterm prognosis rates.

### Study Limitations

This is a single-arm observational study with a limited population number, and, subsequently, the events rate is limited for statistically robust results. Another limitation is the absence of a control group of PVL patients who had not received transcatheter treatment. Furthermore, the absence of a core lab, mainly regarding screening and follow-up echocardiography studies as well as the fact that a part of the data is retrospective, may render our results susceptible to bias. However, the lack of large randomized trials in this field strengthens the role of small observational registries in a field that is going to be increasingly important in the near future, as the total number of symptomatic PVL patients is going to increase. Unfortunately, the initially screened PVL population was not included in the analysis. However, no patient was excluded from transcatheter treatment due to unfavorable anatomical characteristics. A severe frailty index or an active infection were the main reasons for a patient to not receive transcatheter treatment.

## 5. Conclusions

Transcatheter paravalvular leak closure can be performed with high technical and clinical success, irrespective of the prominent indication of the closure or the type of valve that was treated. Despite the relatively high mortality rate during the 24 months of follow-up, the midterm survival rates following transcatheter PVL closure seem to be higher for patients treated in the aortic valve. Most importantly, these procedures should be performed by well-trained and experienced medical teams in carefully selected patients.

## Figures and Tables

**Table 1:** Baseline demographic and clinical characteristics in the total cohort and heart failure/hemolysis subgroups.

	Total (*n* = 60)	Heart Failure (*n* = 24)	Hemolysis (*n* = 36)	*p* Value
Demographics Age (years)	69.5 ± 11	68.5 ± 11.8	70.22 ± 10.8	0.281
Male (%)	39 (65%)	16 (66.7%)	23 (63.9%)	
Female (%)	21 (35%)	8 (33.3%)	13 (36.1%)	
Male versus female				1.000
Body mass index	25.9 ± 2.9	26.6 ± 2.8	25.4 ± 2.9	0.285
Cardiovascular risk factors/medical history				
Diabetes mellitus	11 (18.3%)	4 (16.7%)	7 (19.4%)	0.905
Smoking	10 (16.7%)	6 (25%)	4 (11.1%)	0.254
Chronic kidney disease	21 (35%)	5 (20.8%)	16 (44.4%)	0.011
Previous permanent pacemaker	14 (23.3%)	5 (20.8%)	9 (25%)	0.431
Previous coronary artery disease	14 (23.3%)	7 (29.2%)	7 (19.4%)	0.397
Previous coronary artery bypass	12 (20%)	6 (25%)	6 (16.7%)	0.391
Previous stroke	4 (6.7%)	2 (8.3%)	2 (5.6%)	0.801
Pulmonary hypertension	29 (48.3%)	10 (41.7%)	19 (52.8%)	0.254
Previous Endocarditis	10 (16.7%)	2 (8.3%)	8 (22.2%)	0.149
Atrial fibrillation	30 (50%)	11 (45.8%)	19 (67.9%)	0.201
Logistic EuroSCORE	24.5 ± 13.5	21.02 ± 10.2	27.36 ± 15.3	0.391

**Table 2:** Patient characteristics in mitral/aortic valve subgroups.

	Mitral Valve (*n* = 41)	Aortic Valve (*n* = 19)	*p* Value
Gender			
Male	21 (51.2%)	17 (89.5%)	
Female	20 (48.8%)	2 (10.5%)	
Male versus female			0.003
Cardiovascular risk factors/medical history			
Diabetes mellitus	8 (19.5%)	2 (10.5%)	0.764
Smoking	8 (19.5%)	2 (10.5%)	0.363
Chronic kidney disease	18 (43.9%)	3 (15.8%)	0.016
Previous permanent pacemaker	12 (29.2%)	2 (10.5%)	0.159
Previous coronary artery disease	11 (26.8%)	3 (15.8%)	0.277
Previous coronary artery bypass	11 (26.8%)	1 (5.3%)	0.046
Previous stroke	2 (4.9%)	2 (10.5%)	0.421
Pulmonary hypertension	24 (58.5%)	4 (21.1%)	0.005
Previous Endocarditis	4 (9.8%)	6 (31.6%)	0.049
Atrial fibrillation	23 (56.1%)	7 (36.8%)	0.108
Prominent indication for PVL closure			
Hemolysis	30 (73.2%)	6 (31.6%)	0.034
Heart failure symptoms	11 (26.8%)	13 (68.4%)	0.030
Hemoglobin baseline	9.7 ± 1.3	11.2 ± 1.9	0.006
Risk scores			
Logistic EuroSCORE	26.8 ± 14.5	17.7 ± 8.7	0.024

**Table 3:** Procedural characteristics.

	Total (*n* = 60)	Mitral Valve (*n* = 41)		Aortic Valve (*n* = 19)
Number of devices used				
One (1)	38 (63.3%)	27 (65.8%)		12 (63.1%)
Two (2)	11 (18.3%)	7 (17.1%)		4 (21.1%)
Three (3)	4 (6.7%)	3 (7.3%)		1 (5.2%)
None	4 (6.7%)	2 (4.8%)		2 (10.5%)
Type of device implanted				
Amplatzer vascular plug II	9	5		4
Amplatzer vascular plug III	53	37		16
Occlutech paravalvular leak device	4	2		2
Amplatzer duct occluder	7	7		0
Total number of devices	73	51		22
Fluoroscopy time (minute)	48 ± 25	46 ± 21		53 ± 35
Access site				
Transarterial retrograde	30	15		15
Transeptal anterograde	12	10		2
Transapical retrograde	12	11		1
Transarterial and transeptal	6	5		1
Valve type				
Bioprosthesis	6 (14.6%)		6 (31.6%)	
Mechanical prosthesis	35 (85.4%)		13 (68.4%)	
TAVI	0 (0%)		0 (0%)	

## Data Availability

The data from each center, collected in a single database which anyone can found it after communication with the email: theklalytra@hotmail.com.

## References

[1] Leon M.B., Smith C.R., Mack M.J., Makkar R.R., Svensson L.G., Kodali S.K., Thourani V.H., Tuzcu E.M., Miller D.C., Herrmann H.C. (2016). Transcatheter or Surgical Aortic-Valve Replacement in Intermediate-Risk Patients. N. Engl. J. Med..

[2] Ruiz C.E., Hahn R.T., Berrebi A., Borer J.S., Cutlip D.E., Fontana G., Gerosa G., Ibrahim R., Jelnin V., Jilaihawi H. (2017). Clinical Trial Principles and Endpoint Definitions for Paravalvular Leaks in Surgical Prosthesis: An Expert Statement. J. Am. Coll. Cardiol..

[3] Jilaihawi H., Kashif M., Fontana G., Furugen A., Shiota T., Friede G., Makhija R., Doctor N., Leon M.B., Makkar R.R. (2012). Cross-Sectional Computed Tomographic Assessment Improves Accuracy of Aortic Annular Sizing for Transcatheter Aortic Valve Replacement and Reduces the Incidence of Paravalvular Aortic Regurgitation. J. Am. Coll. Cardiol..

[4] Giblett J.P., Rana B.S., Shapiro L.M., Calvert P.A. (2019). Percutaneous management of paravalvular leaks. Nat. Rev. Cardiol..

[5] Baumgartner H., Falk V., Bax J.J., De Bonis M., Hamm C., Holm P.J., Iung B., Lancellotti P., Lansac E., Rodriguez Muñoz D. (2017). 2017 ESC/EACTS Guidelines for the management of valvular heart disease. Eur. Heart J..

[6] Nishimura R.A., Otto C.M., Bonow R.O., Carabello B.A., Erwin J.P., Fleisher L.A., Jneid H., Mack M.J., McLeod C.J., O’Gara P.T. (2017). 2017 AHA/ACC Focused Update of the 2014 AHA/ACC Guideline for the Management of Patients with Valvular Heart Disease: A Report of the American College of Cardiology/American Heart Association Task Force on Clinical Practice Guidelines. J. Am. Coll. Cardiol..

[7] Joseph T.A., Lane C.E., Fender E.A., Zack C.J., Rihal C.S. (2018). Catheter-based closure of aortic and mitral paravalvular leaks: Existing techniques and new frontiers. Expert Rev. Med. Devices.

[8] Alkhouli M., Sarraf M., Maor E., Sanon S., Cabalka A., Eleid M.F., Hagler D.J., Pollak P., Reeder G., Rihal C.S. (2016). Techniques and Outcomes of Percutaneous Aortic Paravalvular Leak Closure. JACC Cardiovasc. Interv..

[9] Zamorano J., Gonçalves A., Lancellotti P., Andersen K.A., González-Gómez A., Monaghan M., Brochet E., Wunderlich N., Gafoor S., Gillam L.D. (2016). The use of imaging in new transcatheter interventions: An EACVI review paper. Eur. Hear. J. Cardiovasc. Imaging.

[10] Vavuranakis M., Kalogeras K., Lozos V., Aznaouridis K., Aggeli K., Moldovan C., Kalantzis C., Siasos G., Koufakis N., Tousoulis D. (2018). Transapical closure of multiple mitral paravalvular leaks with dual device deployment through a single sheath: A Heart Team job. Hell. J. Cardiol..

[11] Goktekin O., Vatankulu M.A., Ozhan H., Ay Y., Ergelen M., Tasal A., Aydin C., Ismail Z., Ates I., Hijazi Z. (2016). Early experience of percutaneous paravalvular leak closure using a novel Occlutech occluder. Eurointervention.

[12] Onorato E.M., Muratori M., Smolka G., Malczewska M., Zorinas A., Zakarkaite D., Mussayev A., Christos C.P., Bauer F., Gandet T. (2020). Midterm procedural and clinical outcomes of percutaneous paravalvular leak closure with the Occlutech Paravalvular Leak Device. Eurointervention.

[13] Kalogeras K., Ntalekou K., Aggeli K., Moldovan C., Katsianos E., Kalantzis C., Bei E., Mourmouris C., Spargias K., Chrissoheris M. (2021). Transcatheter closure of paravalvular leak: Multicenter experience and follow-up. Hell. J. Cardiol..

[14] Hourihan M., Perry S.B., Mandell V.S., Keane J.F., Rome J.J., Bittl J., Lock J.E. (1992). Transcatheter umbrella closure of valvular and paravalvular leaks. J. Am. Coll. Cardiol..

[15] Alkhouli M., Rihal C.S., Zack C.J., Eleid M.F., Maor E., Sarraf M., Cabalka A.K., Reeder G.S., Hagler D.J., Maalouf J.F. (2017). Transcatheter and Surgical Management of Mitral Paravalvular Leak: Long-Term Outcomes. JACC Cardiovasc. Interv..

[16] Ruiz C.E., Jelnin V., Kronzon I., Dudiy Y., Del Valle-Fernandez R., Einhorn B.N., Chiam P.T., Martinez C., Eiros R., Roubin G. (2011). Clinical Outcomes in Patients Undergoing Percutaneous Closure of Periprosthetic Paravalvular Leaks. J. Am. Coll. Cardiol..

[17] Alkhouli M., Zack C.J., Sarraf M., Eleid M.F., Cabalka A.K., Reeder G.S., Hagler D.J., Maalouf J.F., Nkomo V.T., Rihal C.S. (2017). Successful Percutaneous Mitral Paravalvular Leak Closure Is Associated with Improved Midterm Survival. Circ. Cardiovasc. Interv..

[18] Yang C., Liu Y., Tang J., Jin P., Li L., Yu S., Yang J. (2020). Prognosis of Transcatheter Closure Compared with Surgical Repair of Paravalvular Leak after Prosthetic Valve Replacement: A Retrospective Comparison. Thorac. Cardiovasc. Surg..

[19] García E., Arzamendi D., Jimenez-Quevedo P., Sarnago F., Martí G., Sanchez-Recalde A., Lasa-Larraya G., Sancho M., Iñiguez A., Goicolea J. (2017). Outcomes and predictors of success and complications for paravalvular leak closure: An analysis of the SpanisH real-wOrld paravalvular LEaks closure (HOLE) registry. Eurointervention.

[20] Calvert P.A., Northridge D.B., Malik I.S., Shapiro L., Ludman P., Qureshi S.A., Mullen M., Henderson R., Turner M., Been M. (2016). Percutaneous Device Closure of Paravalvular Leak: Combined Experience From the United Kingdom and Ireland. Circulation.

[21] Hascoët S., Smolka G., Blanchard D., Kloëckner M., Brochet E., Bouisset F., Leurent G., Thambo J.-B., Combes N., Dumonteil N. (2022). Predictors of Clinical Success After Transcatheter Paravalvular Leak Closure: An International Prospective Multicenter Registry. Circ. Cardiovasc. Interv..

